# How to judge pelvic malposition when assessing acetabular index in children? Three simple parameters can determine acceptability

**DOI:** 10.1186/s13018-020-1543-9

**Published:** 2020-01-15

**Authors:** Yi Yang, Daniel Porter, Li Zhao, Xiang Zhao, Xuan Yang, Suxian Chen

**Affiliations:** 1 Department of Pediatric Orthopaedics, The Children’s Hospital, Zhejiang University School of Medicine, National Clinical Research Center for Child Health, No.3333, Binsheng Road, Hangzhou, 310052 China; 2 Department of Pediatric Orthopaedics, XinHua Hospital affiliated to Shanghai Jiao Tong University School of Medicine, No. 1665, Kongjiang Road, Shanghai, 200092 China; 3 Department of Orthopaedic Surgery, First Hospital of Tsinghua University, No. 6 JiuXianQiao No. 2 St, Chaoyang District, Beijing, 100016 China; 4 Ying-Hua Medical Group of Children’s Bone and Joint Healthcare, Room 16-3103, Lane 133 Linping Road, Hongkou District, Shanghai, 200086 China; 5 Department of Radiology, XinHua Hospital affiliated to Shanghai Jiao Tong University School of Medicine, No. 1665, Kongjiang Road, Shanghai, 200092 China

**Keywords:** Developmental dysplasia of the hip, Digital reconstructed radiographs, Measurement error

## Abstract

**Background:**

The acetabular index (AI) is the most commonly used parameter for diagnosing hip dysplasia. Pelvic malposition can result in misinterpretation of AI measurement especially in younger children. We aimed to investigate the correlation between pelvic orientation and acetabular index (AI) by using digital reconstructed radiographs (DRRs) and identify reliable parameters predictive of pelvic orientation on plain radiographs.

**Methods:**

We retrospectively identified 33 children (52 hips) who received dual source CT examinations. Virtual pelvic models were reconstructed after scanning. After orientating in the standard neutral position, the models were rotated and tilted around corresponding axes. DRRs were generated at every 3° during the process. The acetabular index, the horizontal diameter (Dh) and vertical diameter (Dv) of bilateral obturator foramina, the vertical distance (h) between upper border of pubic symphysis, and Hilgenreiner’s line were measured on each DRR by two independent observers. Rotation index (Rr = right Dh/left Dh), tilt index (Rt = h/Dv), intra-observer error, and inter-observer error of AI were calculated.

**Results:**

For tilt and rotation up to 12.0°, AI increased with anterior tilt and decreased with posterior tilt. And for rotation, it increased on the side toward which the pelvis rotated and decreased on the opposite side. AI varied dramatically if angulation exceeded 6.0°. Malposition below this limit demonstrated the intra- and inter-observer errors were ± 2.0° and ± 3.0° respectively and caused no significant effect on AI measurement.

**Conclusions:**

For children up to age 6 years, an acceptable pelvic plain radiograph can be determined when Rt is approximately between 0.9 and 1.4 and Rr between 0.7 and 1.5. For the first time, we have identified parameters derived from a group of subjects which can predict this degree of malposition. The parameters obturator diameters (Dh), obturator height (Dv), and distance (h) between symphysis and Hilgengreiner’s line can be feasibly measured on X-ray and employed in clinical practice to assess the acceptability of the pediatric pelvic radiograph prior to measurement of the AI.

## Background

Developmental dysplasia of the hip (DDH) is one of the most common limb deformities in children, with an incidence of 1 to 35% depending on age and diagnostic method [1]. Early diagnosis and concentric reduction are the essential elements of successful treatment, thereby increasing the chance of a functional and anatomical hip joint [2]. Radiographical assessments in combination with medical history and clinical examination are assumed to make the diagnosis of DDH [3], among which a plain anteroposterior radiograph of the pelvis is the most frequently used due to low cost, limited radiation, and repeatability [4]. The acetabular index (AI) is the most commonly used parameter for diagnosis. This is defined as the angle between a line through the triradiate cartilage (Hilgenreiner’s line) and a line tangentially connecting the inferior margin of the iliac bone and the superolateral part of the acetabular bony rim [5]. However, a plain radiograph cannot always be of reliable standard because of pelvic malposition during the acquisition of radiograph as well as inaccurate centralization of the X-ray beam [6–8]. In addition, the imprecise location of the superolateral part of the acetabulum may contribute to the measurement error [9]. The variance of AI measurement and pelvic orientation is greater in younger children who are incapable of cooperation during the examination. The reliability and reproducibility of AI measurements have been questioned over many decades [10–12]. If an accurate AI measurement could be made from a non-standard pelvic radiograph, this would be a significant gain in the utility of the measurement [13, 14].

The pelvic motion could be projected into three directions: left-right inclination on the coronal plane, anteroposterior pelvic tilt on the sagittal plane and rotation around the craniocaudal axis [7]. Previous investigations about the influence of pelvic orientation on AI measurement had involved clamping adult cadaveric specimens at different degrees of pelvic tilt and rotation [13]. Since left-right inclination occurs perpendicular to the x-ray, it cannot affect the measurement of AI.

**Fig. 1:**
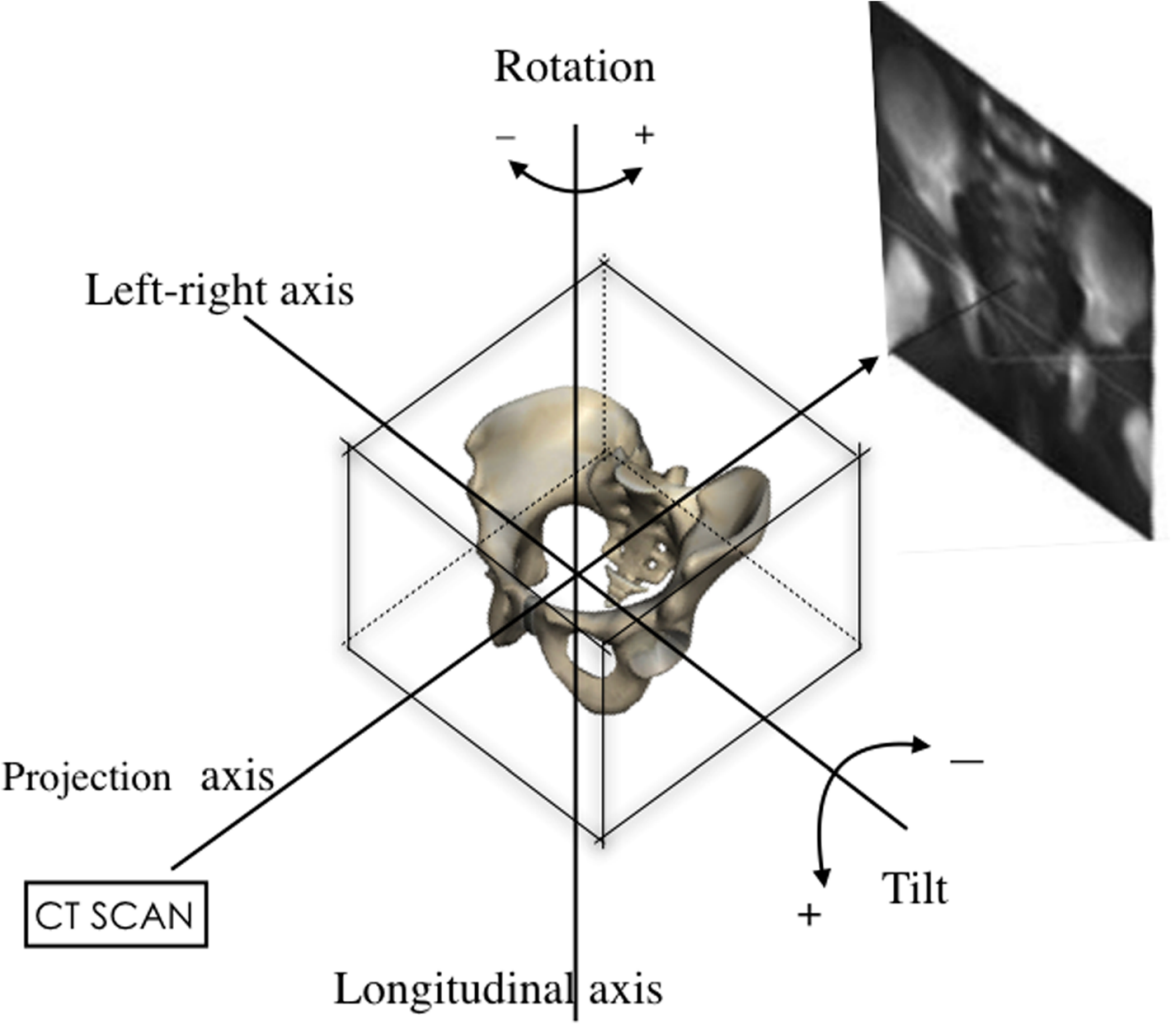
Digital reconstructed radiographs (DRRs) can be generated after tilting and rotating the reconstructed pelvic model to any position. We define the anterior tilt or left rotation as positive

Small sample size, especially in children, is a criticism of the representativeness and reliability of these studies on AI measurement. Recent imaging software advances may now allow resolution of these problems by the use of three-dimensional reconstruction technology based on CT datasets. The reconstructed virtual pelvic model can be subjected to inclination, tilt, and rotation to various degrees (Fig. 1). The accuracy of these digital reconstructed radiographs (DRRs) generated from the virtual pelvic model has been verified previously [15, 16] and these images are shown to be representative of traditional plain radiograph of pelvis [14]. By using DRRs, we are able to expand sample size and to model live, not cadaveric pelvises.

The aims of our study are to (1) investigate the correlation between pelvic orientation and AI measurement by using DRRs generated from pelvic reconstruction models, (2) find relatively reliable parameters on plain radiograph predictive of pelvic orientation, and (3) identify a window of acceptable angulation within which a measurement of AI can be categorized as reliable.

## Methods

We retrospectively reviewed children who had undergone CT examinations for suspected hip dysplasia or pre-operative examination of orthopedic surgery including closed reduction or open reduction/innominate osteotomy in our department between January 2015 and December 2015 from our institutional database. Bilateral hips were counted separately. Inclusion criteria were the availability of dual-source CT data taken via a standardized protocol. Exclusion criteria were (I) subluxated and dislocated hips (the medial beak of the femoral metaphysis lay outside of the lower, inner quadrant formed by the junction of Perkin and Hilgenreiner lines); (II) previous pelvic osteotomy; (III) children over age 8 years due to difficulties in defining Hilgengreiner’s line accurately. We identified 177 patients who received CT examinations and only 33 children (52 hips) met the criteria. In total, 8 boys and 25 girls were included. Among them, 19 children (38 hips) in the out-patient department received CTs for a definite diagnosis of hip dysplasia, one boy (1 hip) received CT for closed reduction and 13 patients (13 hips ) for preparation before innominate osteotomy surgeries.

**Fig. 2:**
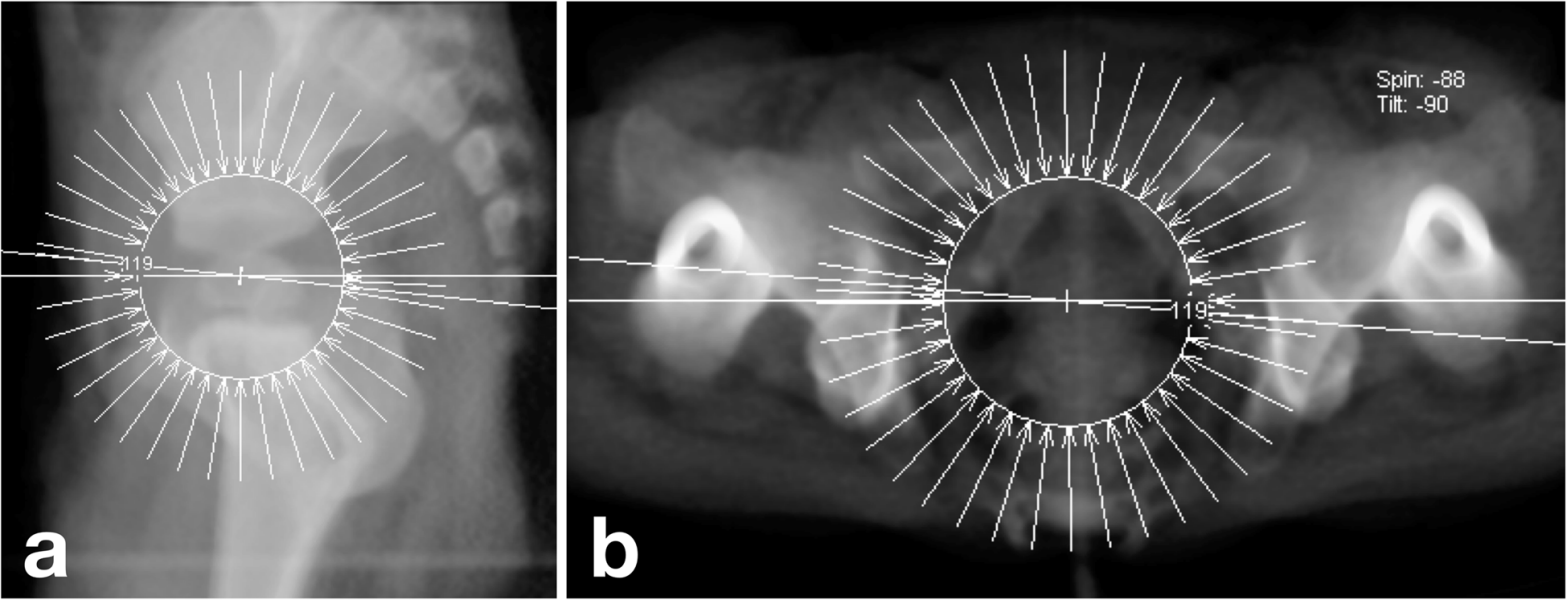
**a** Axis of tilt. **b** Axis of rotation

According to CT protocol, children were placed supine on the scan table after sedation. Each child was carefully placed with body parallel to the longitudinal axis of the table. Legs were extended and aligned in neutral so that both patellae faced the ceiling. The scanning area was from the anterior superior iliac spine to the upper 1/3 of the femur. Each case was scanned by the use of dual-source CT (Siemens, Somatom Definition). Other fixed scanning parameters included slice thickness (0.75 mm), increment (0.5 mm), pitch (0.625 mm), tube voltage (120 kv), and current (33~190 mA). In the post-processing workstation, a virtual pelvic model was reconstructed by using the multiple planar reconstruction (MPR) and volume rendering technique (VRT). After being placed in a standard neutral position, the model was virtually rotated and tilted around its corresponding axes. At the workstation, DRRs were generated at every 3.0° increment. Standard neutral position was defined when both obturator foramina were symmetrical and the anterior pelvic plane (formed by a triangle of pubic symphysis and bilateral anterior superior iliac spines) was parallel to the coronal plane. The tilt axis was defined as a transverse line connecting the center of the left and right triradiate cartilages (Fig. 2a). The rotation axis was defined as a longitudinal line passing through the midpoint of the projected pubic symphysis and sacrococcygeal joint (Fig. 2b).

**Fig. 3:**
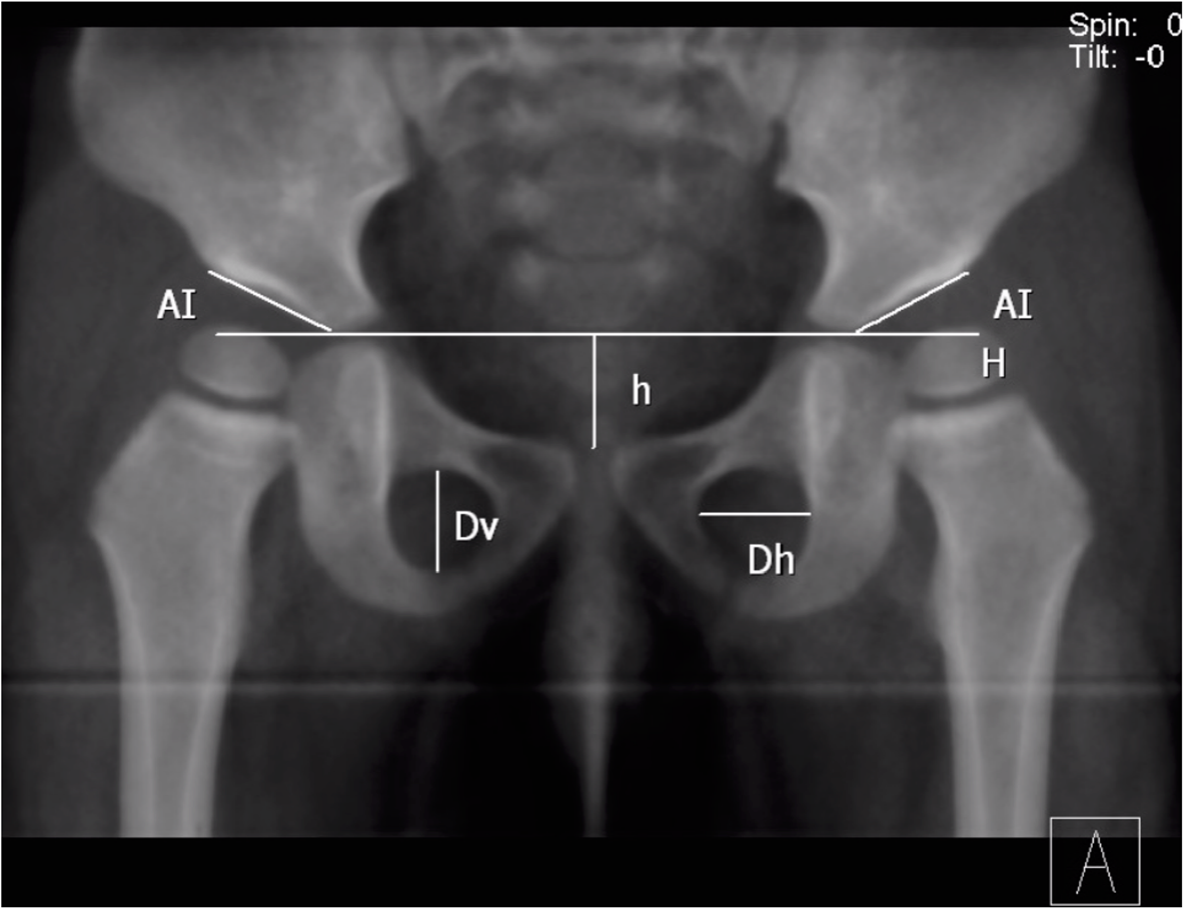
The pelvic DRR of a 1.5-years-old girl and measuring parameters

The acetabular index, the horizontal diameter (Dh), and vertical diameter (Dv) of both obturator foramina, and the vertical distance (h) between the upper border of pubic symphysis and Hilgenreiner’s line were measured on each DRR using our institution’s PACS system (Fig. 3). Rotation index (Rr = right Dh/left Dh) and tilt index (Rt = h/Dv) were then also calculated. These parameters were evaluated by two independent observers (A, B). Observer A evaluated radiographs blind to previous measurements 3 times with a time interval of more than 2 weeks. The intra-observer error was determined by the difference between the readings of observer A from the mean of those readings. The inter-observer error was determined by the difference between the readings of observer B from the mean readings of observer A. AI variance was defined as the difference between the readings measured at other pelvic position from the readings at standard neutral position taken by observer A. A positive AI variance meant an increase of AI value and a negative one meant a decrease. Errors and AI variance were expressed as mean ± SD and the average value of Dh, Dv, and h were used to calculate Rt and Rr. We used SPSS 19.0 software to derive statistics. For continuous variables, the differences between groups were assessed by using an independent sample *t* test. The relationships between different variables were assessed by Pearson correlation analysis. The significance level was set at 5%.

## Results

In the subject group of 33 children (52 hips), the average age was 2.9 ± 1.4 (1.4~6.0) years. Among these, 26 hips were normal and the other 26 hips were dysplastic.

**Fig. 4:**
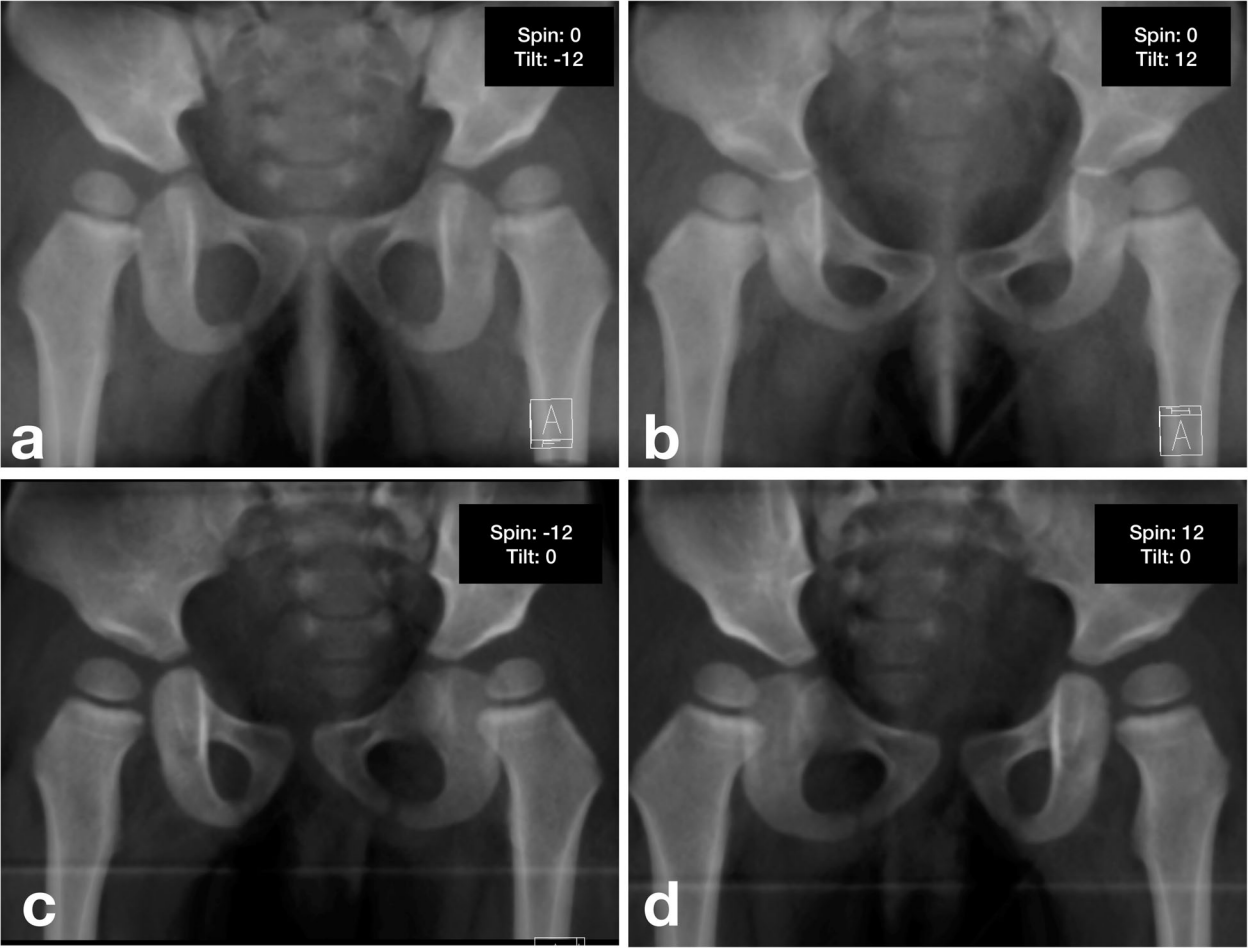
DRRs generated at different pelvic positions (**a** Posteriorly tilt to 12.0°, **b** anteriorly tilt to12.0°, **c** Rotated to right 12.0°, **d** Rotate to left 12.0°)

In total, 120 tilt and 120 rotation DRRs were generated for each case. Interestingly, when pelvis tilted and rotated extended beyond 12.0° from neutral, the superolateral edge of the acetabulum became blurry and hard to be identified. Additionally, we could rarely accept an extremely displaced pelvis in clinical practice for decision-making. Thus, our study focused on images of pelvic tilt and rotation within this range (Fig. 4).

**Fig. 5:**
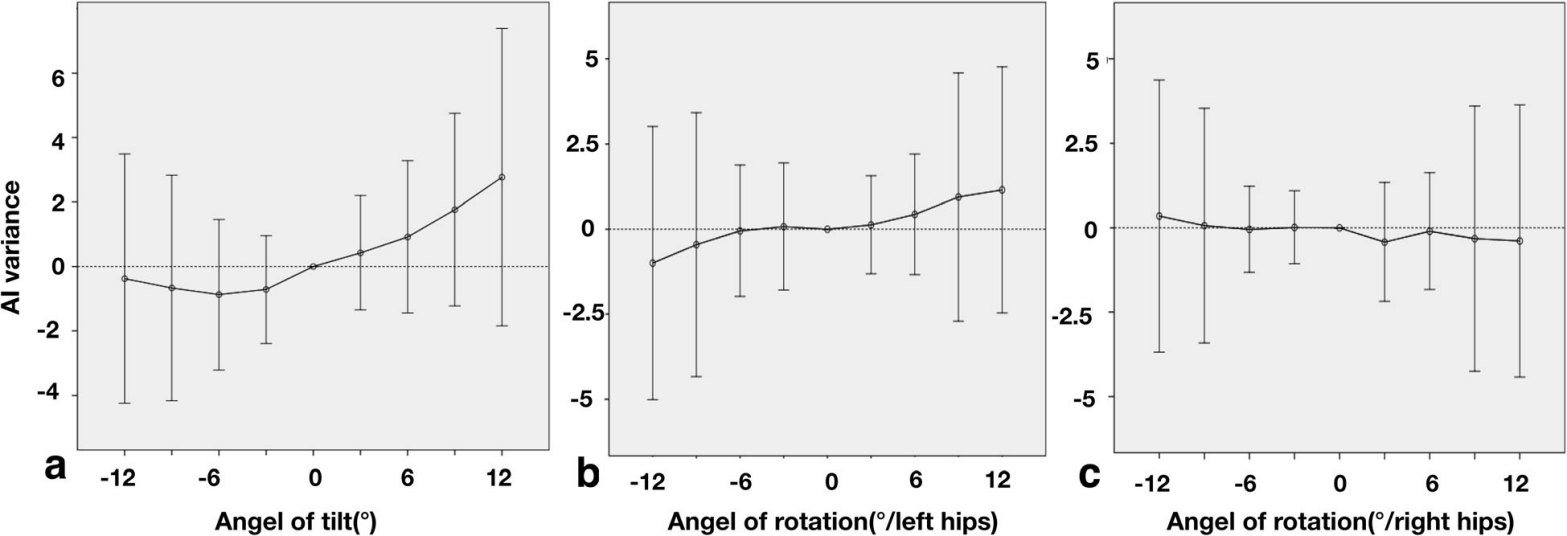
**a** AI variance with pelvic tilt. **b** AI variance with pelvic rotation (left hips). **c** AI variance with pelvic rotation (right hips)

According to Fig. 5, in general, AI variance tended to increase with anterior tilt and decrease with posterior tilt. For rotation, it increased when the pelvis was rotated towards the hip being assessed and decreased on the opposite side. Apparently, this trend cannot be applied to every angle of pelvic orientation since AI variance within 6.0° of rotation showed unpredictability. AI variance changed only slightly and was associated with minor fluctuations in AI standard deviation (SD). The SD of AI variance increased with the increasing angle but it remained less than 2.0° within 6.0° angulation, beyond which the AI value varied in a wider range (Fig. 5).

The irregularity of AI variance reflects the influence of measuring errors. As demonstrated, the intra-observer error of AI was within ± 1.5° and the inter-observer error ± 2.5° when pelvis tilted posteriorly. Compared to anterior tilt to 9.0°, the intra-observer and inter-observer errors were ± 2.0° and ± 3.0° respectively (Table 1). For rotation within 12.0°, the intra-observer error was determined to be ± 2.0° and the inter-observer error ± 2.5° (Table 2). Furthermore, we analyzed the difference in measuring errors between normal and dysplastic hips using *t* tests, the intra-observer error in normal hips was less than that in dysplastic hips. These differences induced by a non-anatomical acetabulum were statistically significant (Table 3).

**Table 1:** The intra- and inter-inter-observer error and the effects caused by pelvic tilt

Pelvic tilt/°	Intra-observer error*	Inter-observer error*	AI variance*	Total measurement error#	AI variance#	Effect
− 12	− 0.1 ± 0.9	− 0.1 ± 2.4	− 0.4 ± 3.9	6.2, − 6.6	7.2, − 8.0	Yes
− 9	− 0.1 ± 0.9	0.1 ± 1.2	− 0.7 ± 3.5	4.3, − 4.2	6.2, − 7.5	Yes
− 6	− 0.2 ± 0.9	0.3 ± 1.3	− 0.9 ± 2.3	4.6, − 4.3	3.7, − 5.5	No
− 3	− 0.3 ± 1.0	0.2 ± 1.6	− 0.7 ± 1.7	4.9, − 5.1	2.6, − 4.0	No
0	− 0.1 ± 1.2	− 0.1 ± 2.4	.00	6.8, − 7.2	0	–
3	0.0 ± 1.8	0.0 ± 2.6	0.4 ± 1.8	8.8, − 8.8	3.9, − 3.1	No
6	0.2 ± 1.9	0.1 ± 2.3	0.9 ± 2.4	8.3, − 7.9	5.6, − 3.7	No
9	0.1 ± 1.7	0.0 ± 2.1	1.8 ± 3.0	7.5, − 7.2	7.6, − 4.1	Yes
12	0.1 ± 2.1	− 0.7 ± 3.3	2.8 ± 4.6	10.0, − 11.4	11.8, − 6.3	Yes

^✻^Expressed as Mean ± SD

^#^Expressed as 95% confidence interval

**Table 2:** The intra- and inter-inter-observer error and the effects caused by pelvic rotation

Pelvic rotation/°	Intra-observer error*	Inter-observer error*	Left AI variance*	Right AI variance*	Total measurement error#	Left AI variance#	Effect	Right AI variance#	Effect
− 12	− 0.1 ± 1.7	− 0.0 ± 2.4	0.5 ± 4.0	0.4 ± 4.0	7.8, − 8.0	8.4, − 7.4	Yes	8.2, − 7.6	Yes
− 9	− 0.0 ± 1.6	0.1 ± 1.6	0.1 ± 3.9	0.1 ± 3.5	6.4, − 6.2	7.6, − 7.6	Yes	6.9, − 6.7	Yes
− 6	− 0.1 ± 1.4	0.2 ± 1.7	− 0.1 ± 1.9	− 0.1 ± 1.3	6.1, − 6.0	3.7, − 3.8	No	2.4, − 2.5	No
− 3	0.0 ± 1.3	0.1 ± 2.1	0.1 ± 1.9	0.0 ± 1.1	6.9, − 6.7	3.7, − 3.6	No	2.1, − 2.1	No
0	− 0.1 ± 1.2	− 0.1 ± 2.4	0.00	0.00	6.8, − 7.2	0	–	0	–
3	0.1 ± 1.1	0.3 ± 2.0	0.1 ± 1.4	− 0.4 ± 1.8	6.4, − 5.5	3.0, − 2.7	No	3.0, − 3.9	No
6	− 0.1 ± 1.1	0.2 ± 1.7	0.4 ± 1.8	− 0.1 ± 1.7	5.6, − 5.3	3.9, − 3.0	No	3.3, − 3.5	No
9	0.3 ± 1.2	0.1 ± 2.3	0.9 ± 3.7	− 0.3 ± 3.9	7.2, − 6.4	8.1, − 6.2	Yes	7.4, − 8.0	Yes
12	0.2 ± 1.5	− 0.1 ± 2.0	1.2 ± 3.6	− 0.4 ± 4.0	7.1, − 6.7	8.2, − 5.9	Yes	7.5, − 8.3	Yes

^✻^Expressed as Mean ± SD

^#^Expressed as 95% confidence interval

**Table 3:** Intra- and inter-observer error in normal vs. dysplastic hips

		Errors/°	*t*	*p*(< 0.05)
Intra-observer	Normal	0.8 ± 0.2	− 2.122	0.049
	Dysplastic	1.0 ± 0.2		
Inter-observer	Normal	1.4 ± 0.3	− 0.206	0.840
	Dysplastic	1.4 ± 0.2		

**Fig. 6:**
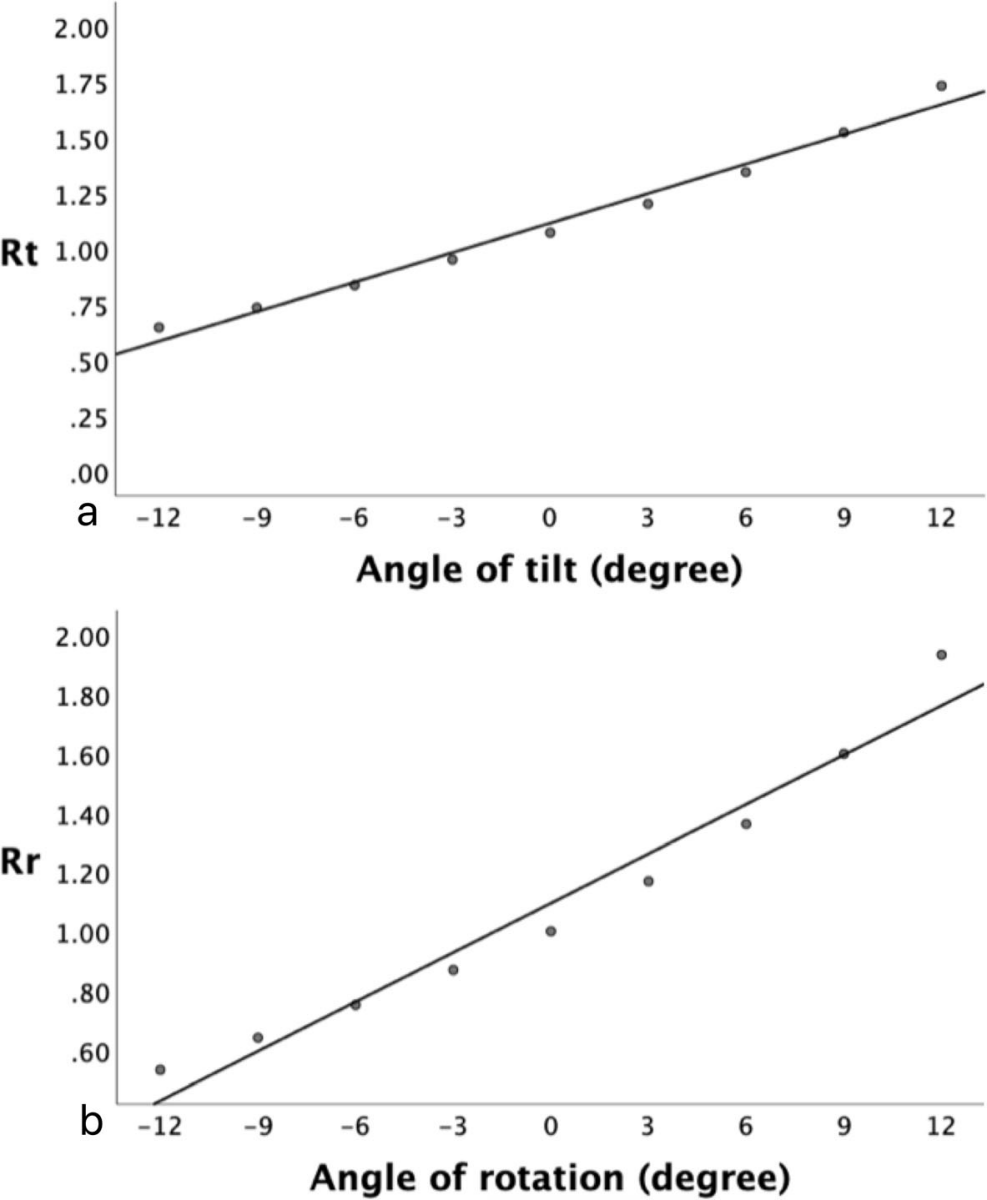
**a** Rt and pelvic tilt. **b** Rr and pelvic rotation

During the simulation, h tended to increase while Dv decreased along with pelvic tilting from − 12.0° to 12.0° and Dh of each obturator foramina decreased on the side towards which that pelvis rotated. As expected, using Pearson correlation analysis, we found a strong correlation between the mimicked tilt angle and tilt index (Rt; *Y* = 1.12 + 0.04*x, *r* = 0.992, *p* < 0.000) and between the mimicked rotation angle and rotation index (Rr; *Y* = 1.09 + 0.06*x, *r* = 0.980, *p* < 0.0001). These findings were established using a model of simple linear regression demonstrated in Fig. 6.

In fact, AI variance is determined by real differences in AI measurement induced by pelvic orientation as well as intra- and inter-observer errors. By definition, if the 95% confidence interval of AI variance is covered by the 95% confidence interval of combined intra- and inter-observer errors, AI variance is most likely to be induced by measurement errors with a probability of 95%. Therefore, changes in pelvic orientation cause no significant effect on AI measurement. As shown in Tables 1 and 2, changes in pelvic orientation did not affect AI measurement within 6.0° of varying pelvic displacement. By using linear regression equations, we found that when Rt is between 0.9 and 1.4 for children younger than 6.0 years of age, pelvic tilt has a minimal effect on AI. Similarly, if Rr is between 0.7 and 1.5, pelvic rotation also causes minimal effect on AI.

## Discussion

In 1936, Kleinberg and Lieberman first described acetabular index as a radiographic method in hip dislocation diagnosis [13] and has since been extensively used in the radiographical evaluation of DDH [17]. It has been used as a predictor for pelvic osteotomy as well as a prognostic indicator after treatment. The plain pelvis radiograph is itself a two-dimensional projection of a three-dimensional structure. Previous researches have proved that malposition of the pelvis can lead to misinterpretation of radiographical statistics including the acetabular index [18, 19]. Reliable quantification of errors caused by malposition in pediatric hip assessments, however, is lacking to date. Our study evaluated these errors by use of DRR images. In addition, we have identified relatively reliable parameters that could be measured on plain radiographs to estimate pelvic orientation.

Tonnis [20] observed that AI increased with pelvic flexion (anterior tilt) and decreased with the extension (posterior tilt), and for rotation that AI decreased on the side towards which the pelvis rotated. In contrast, Portinaro [21] found AI decreased with anterior tilt and increased with posterior tilt. In our study, the results of pelvic tilt were in accordance with those of Tonnis, but not the results of pelvic rotation. Our results suggest that some degree of the variation seen in AI measurement in this and previous studies [6–8] is to be expected due to factors other than malposition. However, in clinical practice, AI value did not always follow the rules of previous studies while our results suggest that the difference in AI measurement is limited and acceptable when pelvic tilt and rotation did not exceed 6.0° since AI variance remains stable and SD of AI variance is always less than ± 2.0° within this window, beyond which AI variance increase dramatically.

In a standard neutral position, our study has identified the intra-observer error to be ± 1.3° and inter-observer error to be ± 2.5° but these errors varied at different pelvic orientation. Spatz [22] calculated the intra-observer error to be ± 3.6° and inter-observer error ± 3.0°. The study of Broughton [23] showed an intra- and inter-observer error of ± 6.1° and ± 5.5° respectively. However, these studies failed to compensate for pelvic malposition. Our calculations of error in neutral position are lower compared to theirs mainly because of the correction of pelvic position. Coincidentally, we discovered that intra-observer error with posterior pelvic tilt was significantly less than when the pelvis shifted in other directions(*p* < 0.000)(Fig. 1). By observing the sequential images of DRRs with increasing posterior pelvic tilt, we found that for most cases, the projection of the superolateral bony rim of acetabulum shifted from indistinct to distinct and then to indistinct again when the pelvis tilted beyond 12.0°. During the process, the anterior and posterior rim of acetabulum tended to overlap as well. We believe that a distinct image of the acetabular superolateral rim in posterior pelvic tilt will reduce errors in AI measurement. In addition, our results revealed a significantly less intra-observer error in the measurement of normal hips compared to dysplastic hips. This difference was not seen in the inter-observer error. This phenomenon that measuring errors could be affected by pelvic orientation and acetabular morphology was not mentioned in previous studies by Skaggs [24] and Borniforti [25]. Portinaro [26] concluded that the error induced by pelvic orientation is only 1.0° if pelvic tilted within 10.0° and rotated within 5.0°. Van der Bom et al. [14] recommended radiographs acquired within ± 4.0° of pelvic rotation and tilt. In comparison to these studies, our experiment revealed that intra-observer error is under ± 2.0° and inter-observer error under ± 3.0° within 6.0° of pelvic displacement.

In adults, sacro-femoral-pubic angle and the vertical distance between the sacrococcygeal joint and the upper rim of pubic symphysis have been proposed as predictors of pelvic tilt, and bilateral iliac width and the horizontal distance between sacrococcygeal joint and the mid-point of the upper rim of pubic symphysis as predictors of pelvic rotation [18, 26–28]. However these parameters cannot be applied to children since the femoral head, sacrum and iliac rim are not completely ossified. Van der Bom et al. [16] used the ratio of vertical distance between sacrococcygeal joint and pubic symphysis to vertical diameter of obturator formamen to evaluate pelvic tilt. However, their data were based on only one cadaveric 3-months-old infant. Actually, we have found it difficult to locate the infant sacrococcygeal joint on plain radiographs. Our study made use of parameters which we consider to be more objective. Derived tilt index (Rt) and rotation index (Rr) show strong correlations with pelvic tilt and rotation respectively. According to our results, the mean Rt of an absolute standard anterior-posterior pelvic X-ray should be around 1.1 for children younger than 6 years. In clinical practice, after applying the angle of 6.0° to the regression equations, a plain radiograph can be considered acceptable if the Rt is approximately between 0.9 and 1.4 and Rr between 0.7 and 1.5. To some extent, our preliminary results provide us with a relatively reliable method based on real patient data. These indices calculated from adopting easily identified bony landmarks could be used as indicators for assessing the acceptability of anterior-posterior pelvic radiographs and contribute to accurate and rapid decision-making in clinical practice.

There are several weaknesses in this study. Acetabular index is an age-related parameter, decreasing gradually in early childhood as the hip develops. The state of ossification and anatomical deformities of the acetabulum will affect the accuracy of AI measurements. These factors are not excluded from the study. We also recognize that pelvic malposition is a combined movement in different directions. Our study represents the effects of single-plane motion, not those of multi-directional movement. Further investigations involving a larger sample size and normal hips are needed for confirmation of these findings.

## Conclusions

Our study made use of DRR images derived from three-dimensional reconstructed pelvises and investigated the correlation between pelvic orientation and AI measurement of children. Within 6.0° of tilt and rotation, pelvic displacement causes no significant effect on AI measurement. In this situation, intra-observer error is below ± 2.0° and inter-observer error below ± 3.0° meanwhile Rt is approximately between 0.9 and 1.4 and Rr between 0.7 to 1.5. For the first time, we have identified parameters derived from a number of subjects which can be applied to predict the degree of malposition. The parameters obturator diameters (Dh), obturator height (Dv), and distance (h) between symphysis and Hilgengreiner’s line can be feasibly measured on X-ray and calculated Rt and Rr could be employed to assess acceptability of the pediatric pelvic radiographs prior to measuring the value of AI and help to minimize the errors in AI measurement.

## Data Availability

All data generated or analyzed during this study are available from the corresponding author on reasonable request.

## Abbreviations

AI: Acetabular index
DDH: Developmental dysplasia of the hip
Dh: Horizontal diameter of obturator foramina
DRR: Digital reconstructed radiograph
Dv: Vertical diameter of obturator foramina
MPR: Multiple planar reconstruction
Rr: Rotation index
Rt: Tilt index
VRT: Volume rendering technique
